# *EHP* Classic Paper of the Year, 2013

**DOI:** 10.1289/ehp.1307395

**Published:** 2013-09-01

**Authors:** Hugh A. Tilson

**Affiliations:** Editor-in-Chief, EHP, E-mail: tilsonha@niehs.nih.gov

*Environmental Health Perspectives* (*EHP*) established the Paper of the Year in 2008 (Tilson 2008) as a way of highlighting high-quality articles published in the journal. The *EHP* Classic Paper of the Year is given to the most highly cited Research Article, Commentary, or Review Article over the preceding 60 months. We are proud to announce that the 2013 *EHP* Classic Paper of the Year is “Effects of Particulate Matter on Genomic DNA Methylation Content and *iNOS* Promoter Methylation” by Letizia Tarantini, Matteo Bonzini, Pietro Apostoli, Valeria Pegoraro, Valentina Bollati, Barbara Marinelli, Laura Cantone, Giovanna Rizzo, Lifang Hou, Joel Schwartz, Pier Alberto Bertazzi, and Andrea Baccarelli, published in the February 2009 issue of *EHP*.

Tarantini et al. (2009) investigated molecular mechanisms underlying health effects of particulate matter exposures using biomarkers of DNA methylation that reflect reprogramming of gene expression. Their 2009 publication was one of the first to report application of epigenetic markers in an environmental or occupational health context.

This research has had a significant influence on human environmental health research. The study by Tarantini et al. (2009) was one of the first studies to report evidence that DNA methylation may be altered in response to short-term exposures, and that it may reflect effects of usual levels of exposure over longer periods of time as well. This study also showed that easy-to-collect biospecimens, such as whole blood samples, and stored archival samples could be used to study effects of exposures on the epigenome. Novel laboratory and statistical approaches used in the study have since become routine in research on the epigenetic effects of environmental agents.

*EHP* congratulates Tarantini and colleagues for their sustained contribution to the environmental health science literature. They should be recognized for their outstanding contribution to the rapidly evolving field of environmental epigenetics.
